# The Effect of Post-Activation Potentiation on Swimming Starts in Adolescent Swimmers

**DOI:** 10.3390/jfmk8020054

**Published:** 2023-05-04

**Authors:** Nikolaos Georgogiannis, George Tsalis

**Affiliations:** School of Physical Education and Sport Science at Serres, Aristotle University of Thessaloniki, 62110 Serres, Greece; nnngeorgog@gmail.com

**Keywords:** post-activation potentiation, swim start, dive, swimming, performance

## Abstract

Background: Post-activation potentiation (PAP) is a phenomenon in which there is an increase in induced momentum in sporting activities after muscle contractions. In swimming, the start of the race and the increase in speed in its first few meters are important. The aim of the present study was to investigate the effect of the PAP protocol, which included a simulated body weight start on the ground, on the swimming start and on the 25 m freestyle performance. Methods: The study included 14 male and 14 female swimmers, 14.9 ± 0.6 years old. All the swimmers performed three maximal attempts of 25 m freestyle swimming from the starting block on three different days in a randomly counterbalanced order. In each session, swimmers performed either a 25 m freestyle without any intervention before the swimming trial (CG), or performed four vertical simulated ground starts at maximal effort, 15 s before (15 sG) or 8 min before (8 minG) the swimming trial. The jump height, entry distance, flight time, and flight speed for each attempt were calculated. Results: The CG entry distance was significantly longer than that of the 15 sG and 8 minG (3.39 ± 0.20 vs. 3.31 ± 0.21 and 3.25 ± 0.25 m, respectively, *p* < 0.001). Conclusions: Four simulated swim starts on the ground, 15 s or 8 min before the swim sprint, had no positive effect on the swim start or swim performance, and it is up to the swimmer to perform these jumps.

## 1. Introduction

**Figure 1:**
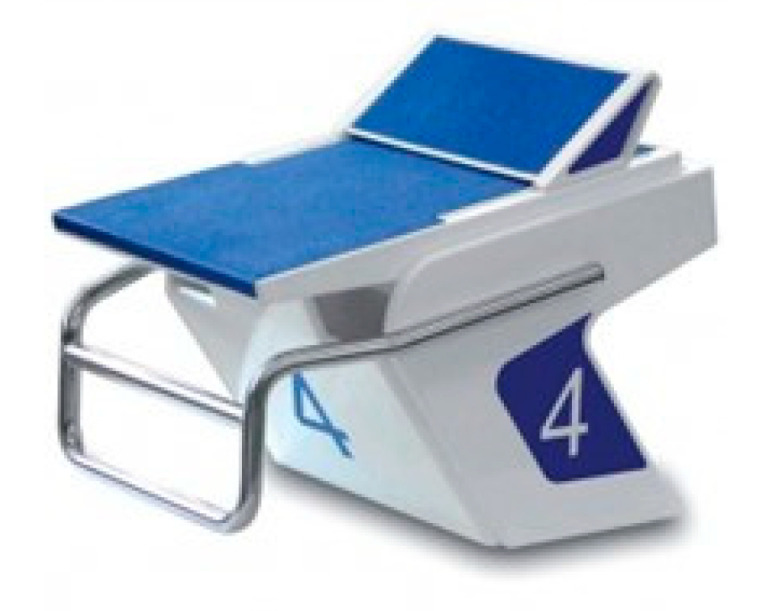
Swimming starting block.

Swimming events, except for the 50 m (m) in an Olympic-size pool, are divided into four main elements: the start, the swim, the turn, and the finish. The freestyle, breaststroke, and butterfly starts are made from a starting platform located on the deck at the edge of the pool, while the underwater start is made from the water with the athletes’ feet in contact with the wall. The start is the process from the sound of an electronic sound device to 15 m, at which point the swimmer’s head must appear, if the swimmer is moving underwater, except for the breaststroke, where the athlete may go beyond 15 m (World Aquatics Swimming Rules (SW 5.3, SW 6.3, and SW 8.3)) [1]. From timing starts in Olympic swimming events, it has been found that the duration of starts is 0.8–26.1% of the total race time, depending on the style and distance of the event [2]. The predominant starting technique used for the freestyle, breaststroke, and butterfly styles is starting with one foot forward and the other foot back on the footrest in the new swimming starting block (Figure 1). The footrest increases the duration of force applied by the athlete on the adjustable kick plate, which results in an increase in horizontal take-off speed [3].

From the above, the importance of the starting phase and the parameters that determine it, such as the reaction time, take-off speed from the starting platform, dive distance, flight speed, and flight time, is obvious. The take-off from the blocks requires explosive force, mainly from the lower body, and training methods to increase this force vary. Preparation is also vital for more efficient diving. Swimmers’ warm-ups before their race is mainly performed in the water. The goal of a warm-up is to optimize the athlete’s performance, which is achieved by increasing body temperature and increasing pulse volume [4]. Between the warm-up and each swimmer’s event, there is a rest or recovery period, the duration of which varies depending on the competition schedule. Research has shown that performing additional exercises on land during the rest period had positive effects on swim starts and swim competitions compared to passive rest [5].

In recent years, the role of post-activation potentiation/facilitation (PAP) [6] in athletic activity has been more extensively investigated, particularly in speed running and jumping. This is because its effects on activities of short duration and explosive force can become apparent a few minutes (min) after its application [7]. PAP, or pre-activation, is defined as a phenomenon in which an increase in elicited torque is observed in various sporting activities after performing minimal voluntary repetitions of maximal or sub-maximal effort [8]. The mechanisms behind post-stimulatory facilitation have not been fully elucidated by the scientific community so far. However, the greater recruitment of motor units and the phosphorylation of myosin light chains are considered to be of predominant importance [9].

The role of PAP has been studied in both professional athletes and recreational trainees. The results of the research and the protocols employed vary. Till and Cooke [10] evaluated the effect of PAP on a 20 m sprint and vertical jumps after applying three different protocols to 20 professional football players. They observed that from 4 min to 9 min, there was no significant increase in the speed or jumping ability compared to the control group. In swimming, the role of PAP has been studied with protocols with different targeting. These include tethered or semi-tethered swimming, lunges, and squats. Research studies, such as those conducted by Sarramian et al. [11] and Hancock et al. [12], have shown improvements in 50 and 100 m freestyle swimming times. In contrast, research conducted by Abbes et al. [13] showed no changes in the 50 m time following the application of a 10 s pre-activation protocol of tethered swimming. Other studies using a countermovement jump (CMJ) as the pre-activation, 8 min before measurement, showed an improvement in the 25 m flutter kick [14].

Finally, studies using upper-body protocols, 8 min before the test, such as simulated out-of-water hand swimming with resistance tubes, showed an improvement in propulsive force during the hand cycle in swimmers, without this improving their performance in the 25 m freestyle swim [15]. Cuenca-Fernández et al. [16] observed an increase in swimmers’ speed, both at 5 and 15 m, 8 min after the two pre-activation protocols that were applied. More specifically, after a swimming warm-up, one group of athletes performed three projections on a Smith machine at 85% of their maximum effort, while another performed four approximate maximum intensity starts on a specially designed platform with pulleys (YOYO). However, in the protocol with four simulated tethered resistance starts, a more significant increase in the athletes’ speed was seen at 5 and 15 m. The PAP effect has been studied on swimming starts by researchers using different protocols, from which confusing results have been obtained. Kilduff et al. [17] evaluated the effects of PAP after 15 s, 4 min, 6 min, 8 min, 12 min, and 16 min of an acute intervention with three barbell squats and a load of 87% of one maximal repetition. They observed an improvement in the athlete’s maximum horizontal and vertical force generated on the starting block. On a specially designed platform, with the protocol of four tethered resistance starts, Cuenca-Fernández et al. [16] showed that the athletes had a higher flight speed, shorter flight time, and longer water entry distance.

Most research uses equipment that athletes cannot access before their race, even more so for swimmers in the limited area of a swimming pool. Still, many times, the time for the athlete to carry out their warm-up before competing is too long, and consequently the effects of PAP are not evident. The concern therefore arises as to whether exercises highly related to the swimming start (such as a vertical jump from the sitting position (jump squat) with body weight alone [18]) or a simulated start (which can be performed at a predetermined time before the athlete’s event) can bring about positive changes in the athlete’s start. Moreover, it has been observed that many jumpers and swimmers sometimes bounce before their event. Therefore, the purpose of the present study was to investigate the effect of performing a simulated ground start, before 25 m of freestyle swimming, on the swimmers’ dives and performance by assuming that the swimmers’ habit of performing jumps before the race gives them a better start than those who do not.

## 2. Materials and Methods

### 2.1. Participants

The necessary sample size (n) was determined with G*Power 3.1.9.7 for Windows (G*power, University of Düsseldorf, Düsseldorf, Germany). Determining a medium effect size at 0.5 for two groups (male and female) and three measurements found that a sample size of 14 participants would be needed to detect significant differences, giving a 95% probability of rejecting the null hypothesis. Twenty-eight competitive swimmers (fourteen male and fourteen female) participated in the study. The athletes were members of different Hellenic swimming teams. All the swimmers participated in the Greek Swimming Championships and their Fina performance points ranged from 345 to 486 for the males and from 397 to 617 for the females for the 50 m freestyle event. All the study participants trained 5–6 times per week and covered an average distance of 5 km in each training session. They also completed dryland training twice a week. Athletes who had recently become ill or had missed more than three consecutive workouts in the previous two weeks for various reasons were excluded from the study. The athletes at the time of the measurements were at the beginning of a preparation cycle after their winter championships. Their age, anthropometric characteristics, competitive experience, best times in the 25 m freestyle, and maximum vertical jump heights are listed in Table 1. The participants were fully informed of the experimental procedures and risks, and written informed parental consent was obtained for each subject, in accordance with the Declaration of Helsinki, before the experiment. The study was conducted in accordance with the rules of the ethics committee of the Aristotle University of Thessaloniki, Serres (ERC-002/2022, 19 January 2022).

### 2.2. Procedures

The swimming pool where the research was conducted was an Olympic-size indoor swimming pool with 8 lanes and an average depth of 2.00 m. The water temperature was 26.5 °C, the air temperature was 25 °C, and the humidity was about 65%. In the swimming pool, there was a large area where the participants performed their warm-ups, and the pool deck was covered with a solid rubber surface for the swimmers’ jumps. Before the final attempt, some preliminaries were conducted and, after adequate rest, the efforts were recorded. The criterion for swimmers to participate was that they were in good condition at the time, had eaten at least three hours before, and had not consumed any energy drinks or coffee.

Participants came to the research site on four consecutive days. On the first day, their age, competitive swimming experience, and best times in the 25 m freestyle were recorded. They also measured their stature with a Seca 213 stadiometer (Seca GmbH & Co. KG., Hamburg, Germany) and their body mass with a Xiaomi Mi Body Composition Scale 2 Smart (XIAOMI Inc., Pekuno, Beijing, China). Finally, the athletes performed three maximal vertical jumps (squat jumps) with a 3 min interval between efforts. Athletes jumped as high as they could and chose their seat depth for the jump [19]. The subjects were familiar with this procedure because they frequently used it in their dryland training sessions. The certified OptoJump system (OptoJump, Microgate, Bolzano, Italy), with a Xiaomi redmi note 9 s quad camera (XIAOMI Inc., Pekuno, Beijing, China), was used to calculate the jumps, fixed on a tripod. With the help of the MyJump2 application [20], the highest of the squat jumps (MaxSJ) was selected.

**Figure 2:**
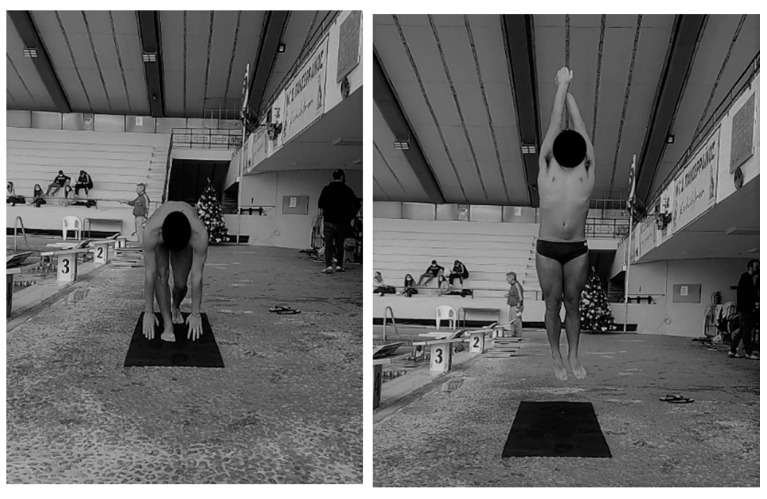
The simulated ground start on the deck.

The swimmers performed three maximum 25 m freestyle attempts with a dive (trial), with different interventions each time before the start and after the same swim warm-up, on three consecutive days with one attempt per day. The warm-up included a 400 m freestyle, a 200 m (50 m backstroke and 50 m breaststroke) freestyle, a 4 × 25 m freestyle with a 1 min kick/pull, and a 2 × 15 m freestyle with maximum effort, all without diving, with a 1 min break between the attempts. The time interval between the warm-up and the 25 m freestyle swim was 15 min. All the participants performed one of the following trials each day in a random counterbalanced order: 25 m effort without any intervention before swimming, designated as the control group (CG); 4 vertical simulated ground starts (SGS4) with maximum effort 15 s before the swim trial (15 sG); SGS4, 8 min before the swim trial (8 minG), using the arms from ground contact, coming into a hydrodynamic position (streamline) during the swing phase (Figure 2).

Athletes performed a 25 m freestyle swim in the seventh lane. A Go Pro 7 camera with a sampling rate of 60 Hz (GoPro Inc., San Mateo, CA, USA), positioned 1 m from the side of the pool in the middle of the first 5 m and at a height of 1 m from the water surface, to fully cover the diving process, was used to video record all the athletes’ attempts. Before the trials, a floating wand with a length of 2 m was positioned in the middle of the lane, from 2 to 4 m from the wall. The length of the wand in pixels (as it was measured using screen coordinates) was used to provide the analogy of screen length to actual length. This analogy was used to transform the screen length to the actual length for the selected variable.

The athletes’ times (T25) for the 3 attempts were recorded using a hand-held Amila Professional stopwatch JS510 (ELDICO SPOR MON. AEE, Athens, Greece). The timing was performed by three official timekeepers, and the median performance was recorded. For the validity of the measurements, timing was stopped when the athlete’s head passed the 25 m point, as it is the sharpest mark. From the video analysis of the dive with Kinovea software (ver. 0.9.5, Kinovea open-source project) [21], the distance to water entry (DE), the flight time (FT), and the average horizontal flight speed (FS) were calculated. The DE was measured from the edge of the starting platform to the point where the fingers of the hand touched the surface of the water. The time calculated from the moment the toes left the edge of the block until the fingers of the hand touched the water surface was FT. FS was calculated from DE and FT. The results were verified by a trustworthy analyst after a randomized control was performed. Finally, the athletes were asked to rate their simulated ground starts (RPE_SGS4_) and dives (RPE_start_) in the 25 m swimming trial on a 10-point BORG scale [22].

### 2.3. Statistical Analysis

Results are presented as the mean ± standard deviation. The normal distribution of the data was checked using Shapiro–Wilk’s test, whereas Levene’s test was used to test the homogeneity of the variance. A two-way analysis of variance (gender × trial) with repeated measures was used to compare SGS4, T25, DE, FT, FS, RPE_SGS4_, and RPEstart. Significant differences were followed up with simple contrasts. The correlation between parameters, independent of gender and trials, was tested by Pearson’s correlation statistic. The Statistical Package for Social Studies SPSS (v27.0, SPSS Inc., Chicago, IL, USA) was used for statistical analysis. The statistical significance was set at α = 0.05.

## 3. Results

The male swimmers were bulkier, faster in the 25 m freestyle, and jumped significantly higher in the vertical jumps than the females (Table 1). During the experimental procedures, swimmers in the CG, 15 sG, and 8 minG trials achieved 96.3 ± 1.4%, 95.5 ± 4.0%, and 95.9 ± 3.2%, respectively, of their best performance in the 25 m freestyle. For the simulated ground start jumps, the swimmers achieved 87.9 ± 19.1% in the 15 sG and 89.5 ± 16.6% in the 8 minG trials for their maximum vertical jump. The results of the trials’ parameters are presented in Table 2. The analysis revealed that there was no gender effect on the trial interaction (F_(1,26.0)_ = 0.105, *p* = 0.749) and there was also no main effect on the trial (F_(1,26.0)_ = 0.444, *p* = 0.511), but there was a main effect of gender on SGS4 only (F_(1,26.0)_ = 8.132, *p* = 0.008). Males jumped higher than females at both 15 sG and 8 minG (5.4 ± 3.2 and 5.8 ± 2.1 cm, *p* = 0.024 and 0.004, respectively).

There was no gender effect on the trial interaction (F_(2,48.6)_ = 0.207, *p* = 0.814) and there was no main effect on the trial (F_(2,48.6)_ = 0.726, *p* = 0.488), but there was a main effect of gender on T25 only (F_(1,26.0)_ = 46.853, *p* < 0.001). Males were faster at CG, 15 sG, and 8 minG for 1.31 ± 0.1, 1.43 ± 0.4, and 1.39 ± 0.2 s, respectively *(p* < 0.001 for all differences). A significant interaction effect, the effects of gender on the trial (F_(2,48.6)_ = 4.060, *p* = 0.023), and the main effects of the trial (F_(2,48.6)_ = 13.788, *p* < 0.001) and gender (F_(1,26.0)_ = 20.649, *p* < 0.005) were found for the DE. Males entered the water as CG for the 15 sG and 8 minG (14 ± 0.3 cm, *p* < 0.001 and 13 ± 0.4 cm, *p* = 0.005, respectively). Females entered the water at a greater distance in the CG and 15 sG than the 8 minG (14 ± 0.5 cm, *p* = 0.003 and 13 ± 0.0 cm, *p* = 0.011, respectively). Males dived significantly further than the females in all the trials (0.39 ± 0.0 cm, *p* < 0.001; 0.26 ± 0.2 cm, *p* = 0.003; and 0.40 ± 0.1 cm, *p* < 0.001, respectively).

For FT, there was no interaction effect of gender on the trial (F_(2,45.6)_ = 0.619, *p* = 0.542) and there was no main effect on the trial (F_(2,45.6)_ = 3.019, *p* = 0.057), but there was the main effect of gender (F_(1,26.0)_ = 5.325, *p* = 0.029). After leaving the starting block, for the CG and 8 minG, the males were in the air for a much more extended period of time than the females in the matched groups (0.6 ± 0.1 s, *p* < 0.013 and 0.6 ± 0.3 s, *p* = 0.043, respectively). For FS, there was no interaction effect of gender on the trial (F_(2,48.7)_ = 0.594, *p* = 0.556) and there was no main effect of the trial and gender (F_(2,48.7)_ = 1.895, *p* = 0.161 and F_(1,26.0)_ = 1.754, *p* = 0.197, respectively). RPE_SGS4_ and RPE_start_ were not significantly different between the trials or genders (*p* > 0.05).

A correlation analysis of the variables showed that the swimmers with a higher MaxSJ performed at a higher SGS4 (r = 0, 634, *p* < 0.001), had a faster T25 (r = −0.590, *p* < 0.001), had a higher DE (r = 0.383, *p* < 0.001), and had a greater FS (r = 0.437, *p* < 0.001) during the dive. Moreover, swimmers who performed at a higher SGS4 had a faster T25 (r = −0.539, *p* < 0.001) and a higher DE (r = 0.502, *p* < 0.001). Moreover, the swimmers who had a higher DE and higher FS swam faster (r = −0.585, *p* < 0.001 and r = −0.234, *p* = 0.032, respectively). Finally, the swimmers who were most fatigued in the simulated ground starts (RPE_SGS4_) felt that it was more difficult to dive (RPE_start_) (r = 0.381, *p* = 0.004).

## 4. Discussion

The aim of the present study was to investigate the effect of performing a simulated ground-based start before 25 m of freestyle swimming on swimmers’ diving and performance. This study investigated whether simulated bodyweight ground starts prior to a swimming start could act as a PAP. Three trials were conducted, where two trials were preceded by simulated ground starts and one trial had no intervention (control group). Specifically, we tested whether four simulated ground starts (15 s or 8 min before the 25 m freestyle attempt) affected the dive parameters from the swim start block. The distance, flight time, flight speed at dive, and 25 m freestyle swimming time were compared in the experimental conditions. The main findings of this study showed that the longest distance was performed without intervention and higher jumps were positively correlated with faster swimming in the 25 m freestyle.

This study showed that the PAP effect did not occur after the four simulated body weight starts in the 15 s and 8 min attempts before the swim start in adolescent swimmers. This observation agrees with the results of the study conducted by Arabatzi et al. [23], which examined the effect of PAP on SJ performance in preadolescent (10–12 years), adolescent (14–15 years), and adult (20–25 years) males and females, and found that the conditioning stimulus had no effect on SJ performance in adults and young people regardless of gender. The lack of effect—compared to the positive effect found in the study conducted by Cuenca-Fernández et al. [16], where four simulated ground starts with resistance tethering were performed before the start—may be due to the higher tethering load of those athletes during the jumps. Although the present study did not examine the maximum horizontal and vertical force generated by the athlete on the starting block, from research related to these parameters by Kilduff et al. [17], it appears that additional loading (such as the intervention with the three back squat repetitions), i.e., the 87% loading of one maximum repetition, is needed to produce the PAP effect. On the other hand, this explanation contradicts the fact that, in the present study, the swimmers in the control group entered the water further away from those in the experimental group, i.e., the jumps probably overloaded the swimmers, but there was no difference in their performance. Moreover, the comparison of these results to the results of a study on football players show agreement in terms of speed and an apparent lagging behind in jumping ability [10].

With regards to the performance in the 25 m freestyle in this study, it was similar in all conditions. Although the 25 m time was influenced by other variables (apart from the dive), such as the underwater dolphin kick, swimming speed, and stroke rate, it appeared that all these characteristics remained constant and did not affect it. Another reason may have been the familiarity of the swimmers with this experimental procedure, either because it is included in their training on land or from the fact that they often use it before competitions. The result is similar to that of the implementation of a 10 s pre-activation swimming pre-activation protocol, where no changes in the 50 m time were observed [13]. However, it seems that in order to achieve the goal of a warm-up, it is necessary to optimize the athlete’s performance and to ensure that the extra jumps do not tire the swimmer [4,5]. Males at T25 were found to be much faster than females (by 1.36 s), in agreement with the results of the first 15 m (0.95 s) in the study conducted by Tor et al. [24]. The greater time difference between the sexes in the present study may indicate a lack of stamina in females. In our study, the DE was longer (0.33 m) than the entry distance (0.26 m) in the study conducted by Tor et al. [24] which did not specify which part of the body was used to estimate the distance. The FT was consistent with the results of Tor et al. [24], although here we also observed a larger difference in the flight time (0.6 s compared to 0.1 s, respectively) between genders. The lack of female strength was also shown here. There was a lack of gender differences in FS in our study, probably because the average flight speed was calculated relative to take-off speed in the study conducted by Tor et al. [24].

It is worth mentioning that the correlation of the above variables revealed that the swimmers who swam the 25 m freestyle faster were the ones who made higher vertical jumps, had a longer water entry distance, and had a higher flight speed. Therefore, vertical jumps can be used as an easy predictor of performance. The phenomenon of PAP in swimming, specifically in the swimming start phase, needs further investigation. Simple and accessible pre-activation protocols should be investigated for the developmental age group of athletes, who make up the largest percentage of the swimming population, with the aim of maximizing their performance. Limitations of this research centered around the timing of 25 m in a 50 m pool. There would be more accuracy with electronic timing in a 25 m pool. However, we believed that swimmers’ performance would remain the same and become slightly improved in absolute numbers. Moreover, there could be more accuracy with a second camera and calibrated measurement space. It would be useful to further investigate both the number of simulated ground starts and their application time, and whether the way in which the simulated ground start with body weight is performed should be modified from vertical to horizontal.

## 5. Conclusions

In conclusion, this study showed that national-level adolescent swimmers did not show any improvement in swim starts after a PAP stimulus. The present findings indicate that the 25 m performance was similar whether swimmers performed the PAP or not. There may be a need for an individual determination of optimal recovery time or an optimal number of jumps; however, further research is needed on this topic. Finally, four simulated swim starts on the ground, 15 s or 8 min before the swim sprint, were found to not have a positive effect on either the swim starts or 25 m swim performance, and it is up to the swimmer whether or not to perform these jumps.

## Figures and Tables

**Table 1:** Participants’ ages, anthropometric characteristics, competitive experience, records in 25 m freestyle, and maximum vertical jump heights are presented as mean ± standard deviation.

	Age (Years)	Height (cm)	Body Mass (kg)	Competitive Experience (Years)	25 m Freestyle Best Time (s)	Maximum Vertical Jump Height (cm)
Male (n = 14)	14.9 ± 0.6	174.7 ± 5.7	61.7 ± 9.6	5.0 ± 1.0	12.37 ± 0.54	28.6 ± 4.7
Female (n = 14)	14.9 ± 0.5	165.8 ± 5.7 *	53.3 ± 7.6 *	5.1 ± 0.6	13.51 ± 0.68 *	23.3 ± 2.0 *

* Significant gender differences for the same parameter.

**Table 2:** Variables measured for the swimmers of the three trial groups: descriptive values and significance levels in the analysis of variance (ANOVA).

Parameters	Gender	CG	15 sG	8 minG	Gender*Trial *p*	Trial *p*
SGS4 (cm)	M		25.5 ± 7.3 ^≈^	26.0 ± 5.7 ^≈^	0.511	0.749
	F		20.1 ± 4.2	20.3 ± 3.6		
T25 (s)	M	12.79 ± 0.52 ^≈^	12.85 ± 0.40 ^≈^	12.81 ± 0.51 ^≈^	0.814	0.488
	F	14.10 ± 0.66	14.28 ± 0.81	14.20 ± 0.69		
DE (m)	M	3.58 ± 0.20 ^≈#†^	3.44 ± 0.17 ^≈†^	3.45 ± 0.24 ^≈#^	0.023 *	<0.001 *
	F	3.19 ± 0.20 ^#^	3.18 ± 0.25 ^†^	3.05 ± 0.25 ^#†^		
FT (s)	M	0.28 ± 0.07 ^≈^	0.29 ± 0.08	0.28 ± 0.09 ^≈^	0.542	0.057
	F	0.22 ± 0.06	0.24 ± 0.06	0.22 ± 0.06		
FS (m/s)	M	5.03 ± 0.38	4.80 ± 0.41	4.96 ± 0.53	0.556	0.161
	F	4.77 ± 0.42	4.71 ± 0.35	4.79 ± 0.42		
RPE_SGS4_ (n)	M		2.6 ± 0.9	2.6 ± 0.9	>0.05	>0.05
	F		2.8 ± 1.1	2.8 ± 1.1		
RPE_start_ (n)	M	2.9 ± 1.1	3.9 ± 1.6	3.6 ± 1.1	0.228	0.186
	F	3.7 ± 1.4	3.8 ± 1.1	3.6 ± 1.4		

Values are shown as the mean of each trial ± the standard deviation. * Statistical differences. ^≈^ Significant gender differences for the same parameter. ^#, †^ Statistical difference between trials. CG: control group; 15 sG: the group that made four simulated ground starts 15 s before the swim trial; 8 minG: the group that made four simulated ground starts 8 min before the swim trial; SGS4: the height of the four simulated ground starts; T25: 25 m freestyle time; DE: distance of entry into the water; FT: flight time; FS: horizontal flight speed; RPE_SGS4_: the fatigue estimation in the four simulated ground stars; RPEstart: the estimation of start difficulty after the intervention of the four simulated ground starts; M: male; F: female.

## Data Availability

All data are available to interested parties upon request.
